# Activin receptor-like kinase 7 promotes apoptosis of vascular smooth muscle cells *via* activating Smad2/3 signaling in diabetic atherosclerosis

**DOI:** 10.3389/fphar.2022.926433

**Published:** 2022-08-17

**Authors:** Shengchuan Cao, Qiuhuan Yuan, Qianqian Dong, Xilong Liu, Weikang Liu, Xiaoxuan Zhai, Chuanxin Zhang, Han Liu, Mengxiong Tang, Shujian Wei, Yuguo Chen

**Affiliations:** ^1^ Department of Emergency Medicine, Qilu Hospital of Shandong University, Jinan, China; ^2^ Shandong Provincial Clinical Research Center for Emergency and Critical Care Medicine, Institute of Emergency and Critical Care Medicine of Shandong University, Chest Pain Center, Qilu Hospital of Shandong University, Jinan, China; ^3^ Shandong Provincial Engineering Laboratory for Emergency and Critical Care Medicine, Key Laboratory of Cardiopulmonary-Cerebral Resuscitation Research of Shandong Province, Key Laboratory of Emergency and Critical Care Medicine of Shandong Province, Qilu Hospital of Shandong University, Jinan, China; ^4^ The Key Laboratory of Cardiovascular Remodeling and Function Research, Chinese Ministry of Education, Chinese Ministry of Health and Chinese Academy of Medical Sciences, The State and Shandong Province Joint Key Laboratory of Translational Cardiovascular Medicine, Qilu Hospital of Shandong University, Jinan, China

**Keywords:** diabetic atherosclerosis, activin receptor-like kinase 7, vascular smooth muscle cells, apoptosis, Smad2/3

## Abstract

Vascular smooth muscle cells (VSMCs) is a vital accelerator in the late phase of diabetic atherosclerosis, but the underlying mechanism remains unclear. The aim of our study was to investigate whether activin receptor-like kinase 7 (ALK7)-Smad2/3 pathway plays an important role in VSMC apoptosis of diabetic atherosclerosis. It was shown that ALK7 expression was obviously elevated in the aorta of ApoE^−/−^ mice with type 2 diabetes mellitus. Inhibition of ALK7 expression significantly improved the stability of atherosclerotic plaques and reduced cell apoptosis. Further experiments showed that ALK7 knockdown stabilized atherosclerotic plaques by reducing VSMC apoptosis via activating Smad2/3. Our study uncovered the important role of ALK7-Smad2/3 signaling in VSMCs apoptosis, which might be a potential therapeutic target in diabetic atherosclerosis.

## Introduction

Atherosclerosis remains the major pathogenesis of ischemic heart diseases and stroke, which represents a leading cause of death worldwide (Weber and Noels, 2011; Hopkins, 2013; Poznyak et al., 2020). In recent years, the important role of diabetes mellitus as risk factor for atherosclerosis becomes more typical and clear. Coexistence of diabetes sophisticates the incidence, distribution and severity of atherosclerosis, leading to increased vulnerability of plaques and more severe clinical events (Moreno and Fuster, 2004; Yuan et al., 2019; Lien et al., 2021). However, there are no effective therapeutic strategies targeting diabetic atherosclerosis.

It is well-accepted that plaque vulnerability is determined by the collagen, vascular smooth muscle cells (VSMCs), lipid and macrophages. VSMCs, the major component of fibrillar cap, play quite the opposite role in the development of plaque (Bennett et al., 2016; Miano et al., 2021). During the early stage of plaque, less VSMC apoptosis results in their accumulation in the intima and media, in favor of the remodeling of atherosclerotic arteries (Ahmadi et al., 2019). While in the late stage of plaque, increased VSMC apoptosis can give rise to thinner fibrillar cap, immensely influencing plaque stability and causing increased possibility of its rupture (Leskinen et al., 2003; Clarke et al., 2006). Excessive VSMC apoptosis was especially evident in the progression of diabetic atherosclerosis. However, the mechanism underlying VSMC apoptosis remains unclear and needs further exploration.

Activin receptor-like kinase 7 (ALK7), firstly isolated from rat brain as an orphan receptor, belongs to the type I TGF receptor superfamily (Rydén et al., 1996; Tsuchida et al., 1996). It is composed of a transmembrane domain, a serine/threonine kinases domain and a glycine-serine-rich (GS) linker between them, and extracellular ligand domain (Tsuchida et al., 2008). ALK7 possesses serine/threonine kinase activity, and is widely expressed in endocrine tissues, such as islet, adipose, which is also detected in brain, liver, heart (Ibáñez, 2021). The interaction of ALK7 with its ligands is relatively weak and is mainly recruited to form a complex with activin type II receptor (ActRII) and phosphorylated by ActRII, signaling to a series of downstream substrates, including Smads proteins (Greenwald et al., 2003). ALK7, a major modulator of metabolic homeostasis (Ibáñez, 2021), was reported to play an important role in diabetic associated cardiomyopathy, aortic stiffness and retinopathy (Liu et al., 2013; Li et al., 2015; Shi et al., 2020). Moreover, ALK7 exerted its proapoptotic role on various cells in diabetic conditions, such as osteoblasts, retinal pigment epithelial cells (Shi et al., 2020; Cheng et al., 2021). It was also reported that ALK7 acted as a positive regulator of atherosclerosis via activating macrophage, exacerbating plaque vulnerability (Cheng et al., 2021). Whether ALK7 was involved in VSMC apoptosis caused by diabetic atherosclerosis needs further exploration.

In the present study, we fed ApoE^−/−^ mice with high fat diet and inject one low-dose streptozotocin to mimic diabetic atherosclerosis and determined to explore the role of ALK7-regulated VSMC apoptosis on the vulnerability of diabetic atherosclerosis and its underlying mechanism. Our results demonstrated that ALK7 promoted VSMC apoptosis and exacerbated diabetic atherosclerosis via Smad2/3 signaling pathway, providing a new therapeutic target for stabilizing atherosclerotic plaques in diabetes mellitus.

## Materials and methods

### Reagents

Primary antibodies against ALK7 were obtained from Santa Cruz Biotechnology (sc374538, United States). Primary antibodies against Smad2/3 (5678), p-Smad2 (3108), p-Smad3 (9520), Bax (2772) and cleaved caspase-3 (9662) were obtained from Cell Signaling Technology (United States). Primary antibodies against MOMA-2 (ab33451) and α-SMA (ab5694) were obtained from Abcam (United Kingdom). Primary antibodies against Bcl-2 was obtained from Genetex (GTX100064, United States), and β-actin was from Proteintech Group (60008-1, China). Secondary antibodies were purchased from Jackson (United States). Streptozotocin (STZ, S0130) and polybrene were purchased from Sigma-Aldrich (United States). SB431542 was obtained from Selleck (S1067,United States).

### Experimental animals

Male ApoE^−/−^ mice were purchased from Beijing Vital River Laboratory Animal Technology Co. Ltd (China) and housed in an environmentally controlled room at 22 ± 2°C and 50% ± 5% humidity with a 12 h/12 h light/dark cycle with free access to food and water. They were allowed to acclimate one week before performing intraperitoneal glucose tolerance test (IPGTT) and then were randomly assigned to control group (Chow group) and diabetic group (DM group). Mice in the diabetic group were fed with a high fat diet (20% fat, 20% sugar, and 1.25% cholesterol; Beijing HFK Bioscience company, China). After 8 weeks, IPGTT was performed to confirm the appearance of insulin resistance. Those mice showing insulin resistance were intraperitoneally injected with one low-dose STZ (75-80 mg/kg body weight in 0.1 mol/L citrate buffer, pH 4.5). Two weeks after STZ injection, most mice displayed hyperglycemia, insulin resistance, and glucose intolerance (Supplementary Figure S1), as previously reported (Wang et al., 2014). Mice with similar degrees of hyperglycemia and body weight were randomly divided into DM (DM, *n* = 20), DM + nc-shRNA (*n* = 20) and DM + ALK7-shRNA, *n* = 20), and injected with nc-shRNA or ALK7-shRNA lentivirus at a dose of 4×10^9^ ifu per mouse via tail vein. At the end of experiment, mice were sacrificed and aortas were collected for subsequent analysis.

### Cell culture and treatment

Mouse primary VSMCs were purchased from Procell Life Science & Technology Co., Ltd. (China), and maintained in complete Dulbecco’s modified Eagle’s medium (DMEM, Thermo Fisher Scientific, United States), supplemented with 10% fetal bovine serum (FBS, Thermo Fisher Scientific), penicillin (100 U/mL) and streptomycin (100 mg/ml) in humidified air with 5% CO_2_ at 37°C. For high glucose and high palmitate co-treatment, VSMCs were supplemented with 25 mM D-glucose and 0.2–0.4 mM BSA-conjugated palmitate for 24 h (Wu et al., 2007; Weikel et al., 2015). VSMCs were added with 5.5 mM glucose, 19.5 mM mannose (osmotic control) and 0.4 mM BSA for normoglycemic and normal palmitate controls. VSMCs from passages 4-10 were used in all experiments.

### Intraperitoneal glucose tolerance test

After fasted for 12–16 h, a bolus of glucose (2 g/kg) was injected intraperitoneally, and blood samples were collected from tail vein at 0, 15, 30, 60 and 120 min. The glucose level was measured using OneTouch Glucometer (LifeScan, CA). The mean area under the receiver operating characteristic curve (AUC) was calculated for glucose level.

### Quantitative real-time PCR analysis

Total RNA was extracted using TRIzol-based (Thermo Fisher Scientific) RNA isolation kit and quantified by Nanodrop (Agilent Technologies, United States). cDNA was reverse transcribed using the HiScript II Q RT SuperMix for qPCR (+gDNA wiper) kit (Vazyme, China). Quantitative PCR was carried out with the ChamQTM SYBR^®^ qPCR Master Mix (Vazyme). The forward primer sequence of ALK7 was 5′- ATG​CTA​ACC​AAC​GGG​AAA​GAG-3′, and reverse primer sequence of ALK7 was 5′-GGA​AGG​TGC​AGT​GTG​ATA​TTG​T-3′. The forward primer sequence of internal reference 18S RNA was 5′-AGG​GGA​GAG​CGG​GTA​AGA​GA-3′, and reverse primer sequence was 5′-GGA​CAG​GAC​TAG​GCG​GAA​CA-3′. The expression level of ALK7 was normalized to 18S RNA (Sangon Biotech Co., Ltd., China) and calculated using the 2^−ΔΔCt^ method.

### Western blot analysis

Aortic tissues or VSMCs were lysed with RIPA lysis buffer supplemented with protease inhibitor (Beyotime Biotechnology, China). Equal protein was separated by sodium dodecyl sulfate-polyacrylamide gel electrophoresis, and transferred onto polyvinylidene fluoride (PVDF) membrane (Millipore, United States). After blocked, the PVDF membranes were incubated with target primary antibodies overnight at 4°C and secondary antibodies for 1–2 h at room temperature. The membranes were then visualized using the enhanced chemiluminescence reagents with American Image 600 (GE, United States). The density of the immunoreactive bands was analyzed using Image J software (National Institutes of Health, United States).

### Immunohistochemistry

After dewaxed and rehydrated, paraffin sections of aortic root were blocked and incubated with primary antibodies (ALK7; MOMA-2; α-SMA) overnight at 4°C. Then they were incubated with horseradish peroxidase-conjugated secondary antibodies followed by 3,3-Diaminobenzidine (DAB) chromogen detection (DAKO). Finally, the sections were counterstained with hematoxylin and the images were captured using Olympus microscope (Japan). The expression of ALK7 was calculated as percentage of ALK7-positive area to total plaque area with the Image J software.

### *En face* analysis of atherosclerotic plaque stability

The aortas were obtained by stripping from the base ascending aorta to the iliac bifurcation and atherosclerotic plaques were determined by *en face* Oil Red O staining. Hematoxylin and eosin (HE) staining was used to detect the lesion size, the area of which was circled and calculated in conjunction with scale bar by Image J software. The intima/media (I/M) ratio was also calculated. In order to analyze the lipids and collagen in the aortic root, consecutive sections of each mouse were stained with Oil Red O and picrosirius red respectively. The plaque stability was assessed by calculating the vulnerable index according to the ratio of area I (Oil Red^+^ + MOMA-2^+^) to area II (α-SMA^+^ + Collagen^+^) as described previously (Dong et al., 2013).

### Activin receptor-like kinase 7 knockdown and overexpression

Short hairpin RNAs (shRNA) against ALK7 and their corresponding control vectors were constructed by Hanbio Biotechnology Co. Ltd. (Shanghai, China), and the sequences of ALK7 were as followed: 5′-GCT​GTG​AAG​CAC​GAT​TCT​ATC-3′. Then they were transfected into VSMCs with 2 mg/ml polybrene and stable VSMCs were selected with 1 mg/ml puromycin for one week. Immunofluorescent staining and western blot was used to determine the infection efficiency. ALK7 overexpression (ALK7-OE) lentivirus were obtained from Applied Biological Materials Inc. (Nanjing, China) and infected VSMCs in the same way with ALK7 shRNA lentivirus infection.

### Transferase-mediated dUTP nick-end labeling assay

Apoptotic cells were determined by terminal deoxynucleotidyl transferase-mediated dUTP nick-end labeling (TUNEL) staining using *In Situ* Cell Death Detection Kit (12156792510, Roche, Germany). The nuclei were counterstained with DAPI. Images were captured by Olympus fluorescence microscope (Japan). The apoptosis proportion was determined by the number of TUNEL-positive nuclei/ total nuclei in the field.

### Statistical analysis

Data were expressed as mean ± SEMs of three independent experiments for the *in vitro* studies. The statistical difference between two groups or among multiple groups was analyzed respectively by the student’s *t*-test or ANOVA in GraphPad Pro5.0 (GraphPad, CA). Difference was considered to be significant at *p* < 0.05.

## Results

### Activin receptor-like kinase 7 expression was elevated in diabetic mice aortas with atherosclerotic plaques

**FIGURE 1:**
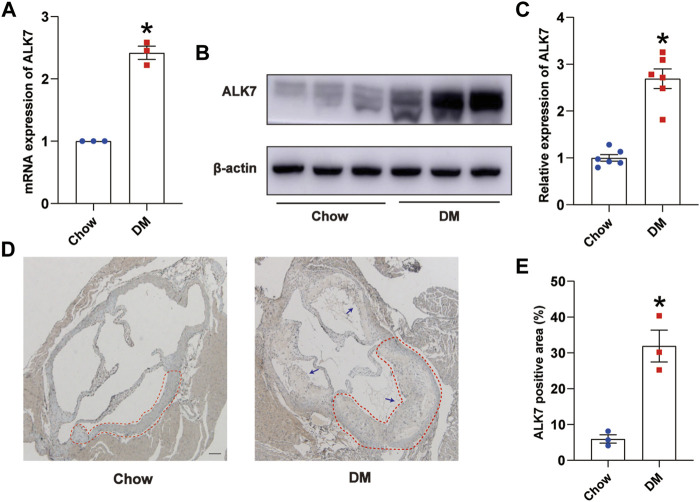
ALK7 was increased in the aortas with atherosclerotic lesions of diabetic mice. **(A)**. Relative mRNA level of ALK7 in the aortas with atherosclerotic plaques of diabetic mice was determined by quantitative real-time PCR. *n* = 3 per group. **(B–C)**. Relative protein level of ALK7 in the aortas with atherosclerotic plaques of diabetic mice was determined by western blot. *n* = 6 per group. **(D–E)**. Immunohistochemical staining of ALK7 was examined in the aortic root and quantified. *n* = 3 per group. Scale bar: 100 μm. Dashed lines encircled some part of atherosclerotic plaque. Arrows indicated all the atherosclerotic plaques in the aortic root. Data was analyzed with student’s *t*-test. *p < 0.05 vs. the Chow group.

In this study, we fed ApoE^−/−^ mice with high fat diet for 8 weeks and then intraperitoneally gave one low-dose injection of streptozotocin to successfully induce type 2 diabetes (Supplementary Figure S1). Results showed that both of the mRNA and protein level of ALK7 were elevated in the aortas with atherosclerotic lesions of diabetic group (Figures 1A–C). Moreover, we found that ALK7 was greatly elevated and extensively distributed in the atherosclerotic plaques of aortic root in the diabetic group (Figures 1D,E). These findings implicated ALK7 acting as a potential regulator in the pathogenesis of diabetic atherosclerosis.

### Activin receptor-like kinase 7 deficiency decreased atherosclerotic plaque burden and increased plaque stability of diabetic mice

**FIGURE 2:**
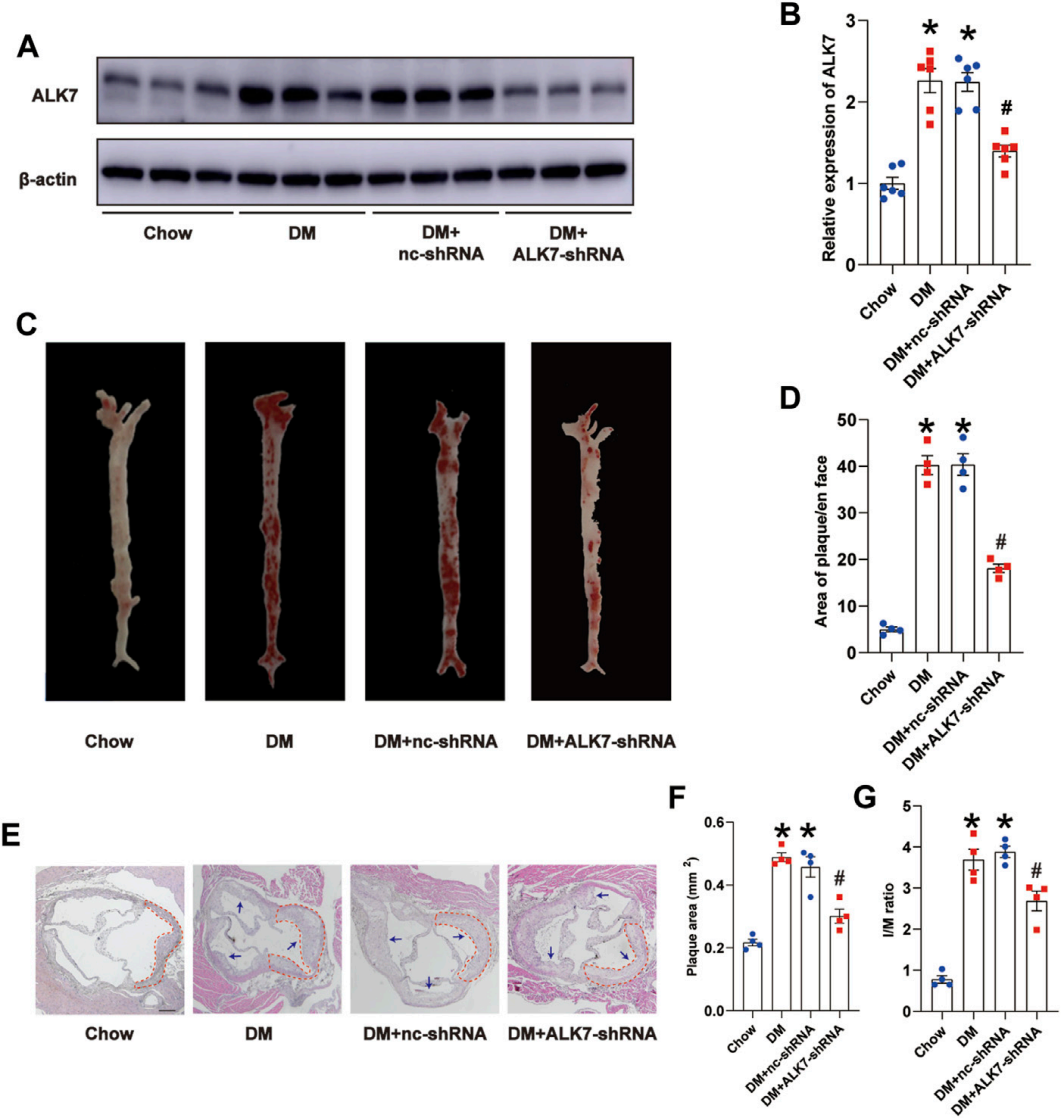
Knockdown of ALK7 reduced atherosclerotic plaque burden of diabetic mice. **(A–B)**. ALK7-shRNA lentivirus was delivered *via* tail vein and the protein level of ALK7 in the aorta was determined by western blot. *n* = 6 per group. **(C–D)**. Representative gross images of atherosclerotic plaques along the aortic trees was examined by the Oil Red O staining. *n* = 4 per group. **(E–G)**. Representative morphological images of aorta roots were stained with hematoxylin and eosin, and the plaque area and I/M ratio was calculated. *n* = 4 per group. Scale bar: 100 μm. Dashed lines encircled some part of atherosclerotic plaque. Arrows indicated all the atherosclerotic plaques in the aortic root. Data was analyzed with ANOVA. *p < 0.05 vs. the Chow group, ^#^ *p* < 0.05 vs. the DM + nc-shRNA group.

Next, we delivered ALK7-shRNA lentivirus *via* tail vein injection to address its precise role on the plaque size and stability. As expected, the protein level of ALK7 was obviously reduced when ALK7 was knocked down (Figures 2A,B). *En face* analysis using Oil Red O staining showed that ALK7 inhibition significantly decreased the number of atherosclerotic plaques along the aortic tree in the diabetic group (Figures 2C,D). Moreover, ALK7 deficiency obviously reduced the increased size of plaque and I/M ratio in the diabetic group (Figures 2E–G). However, ALK7 knockdown exerted no significant influence on glucose and lipid metabolism of diabetic mice (Supplementary Table S1).

**FIGURE 3:**
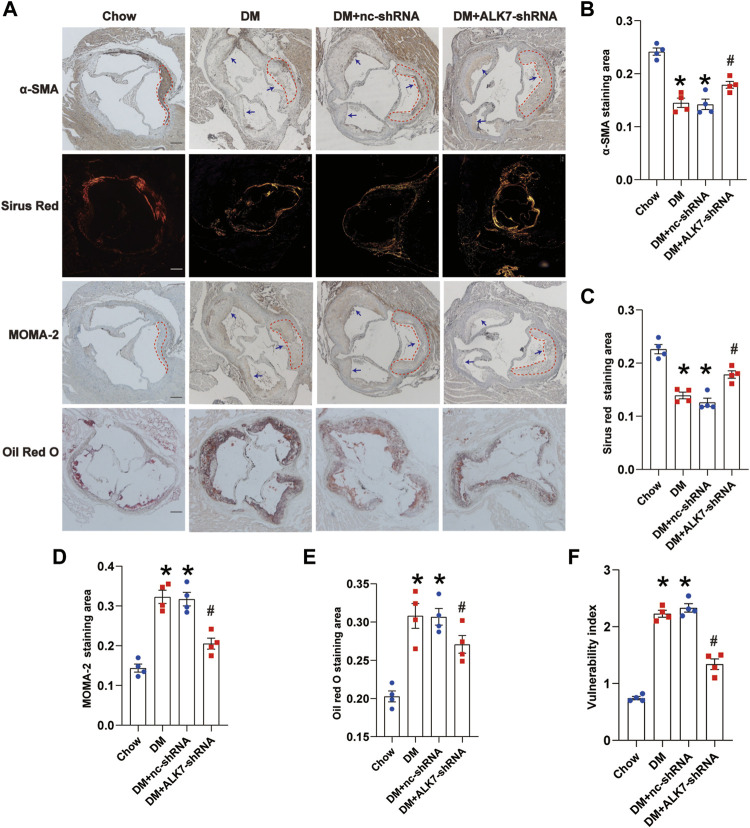
Knockdown of ALK7 increased atherosclerotic plaque stability of diabetic mice. **(A)**. The stability of atherosclerotic plaques in the aortic root was determined by α-SMA for smooth muscle cells, picrosirius red staining for collagen, MOMA-2 for macrophage infiltration, and Oil Red O staining for lipid deposition. *n* = 4 per group. Scale bar: 100 μm. Dashed lines encircled some part of atherosclerotic plaque. Arrows indicated all the atherosclerotic plaques in the aortic root. **(B–E)**. Quantification of smooth muscle cell content, collagen content, macrophage infiltration and lipid accumulation in the atherosclerotic plaques of aortic root. **(F)**. The vulnerability of atherosclerotic plaques was calculated using the ratio of MOMA-2^+^ (%) plus Oil Red (%) to α-SMA (%) plus collagen (%). Data was analyzed with ANOVA. *p < 0.05 vs. the Chow group, ^#^ *p* < 0.05 vs. the DM + nc-shRNA group.

We subsequently examined the effect of ALK7 inhibition on the atherosclerotic plaque vulnerability. It was shown that there were more α-SMA-positive cells and collagen, and less MOMA-2-positive cells and lipids in the atherosclerotic plaque of aorta in the diabetic group with ALK7-shRNA lentivirus delivery (Figures 3A–E). As a result, ALK7 deficiency dramatically decreased the plaque vulnerability index in the diabetic group (Figure 3F).

### Activin receptor-like kinase 7 deficiency improved diabetic atherosclerosis *via* reducing vascular smooth muscle cell apoptosis

**FIGURE 4:**
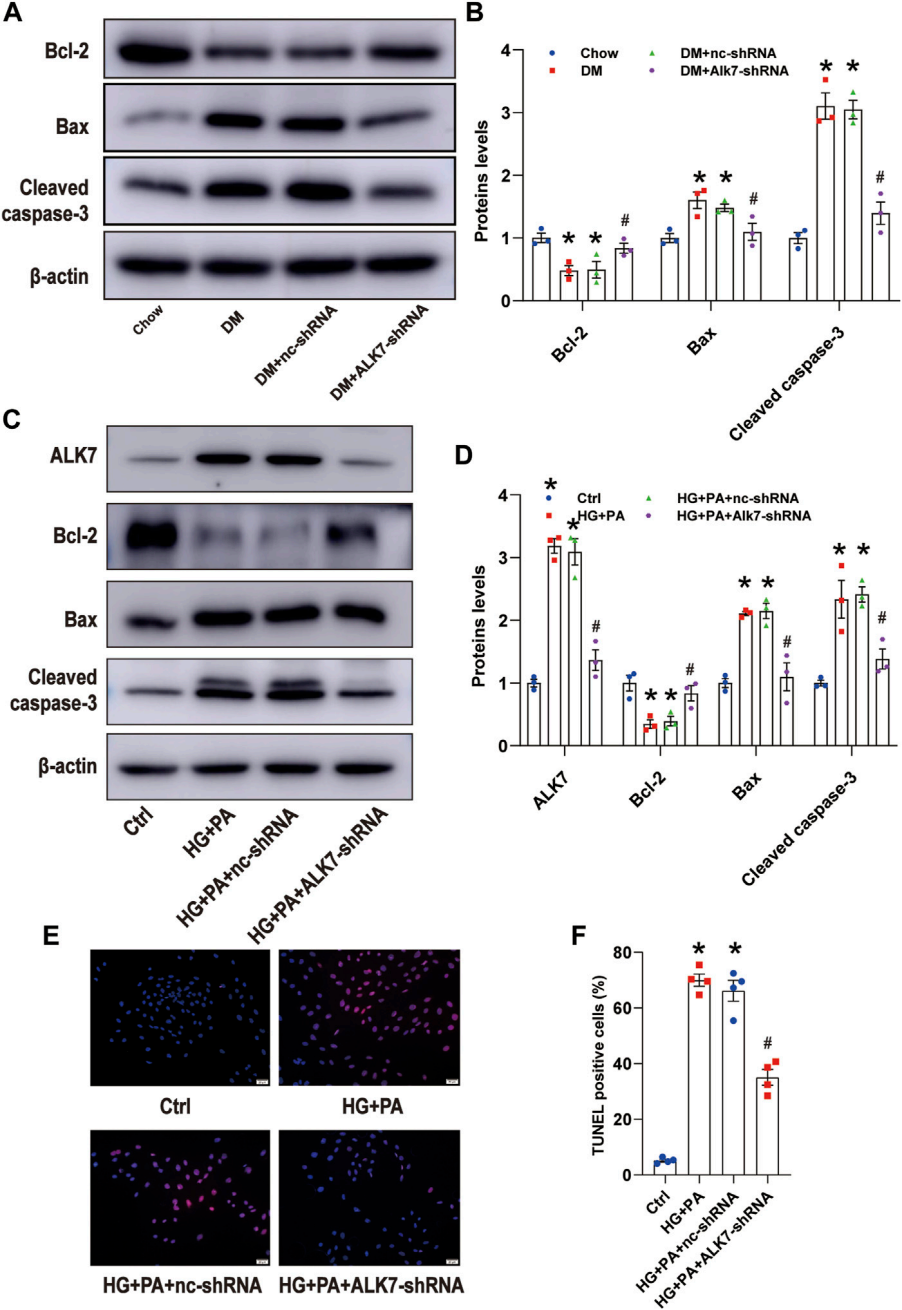
Knockdown of ALK7 inhibited VSMC apoptosis in the atherosclerotic plaques of diabetic mice. **(A–B)**. The protein level of Bcl-2, Bax, and cleaved caspase 3 in the aortas with atherosclerotic plaques of diabetic mice were determined by western blot. *n* = 3 per group. *p < 0.05 vs. the Chow group, ^#^ *p* < 0.05 vs. the DM + nc-shRNA group. **(C–D)**. The protein level of Bcl-2, Bax, and cleaved caspase 3 in VSMCs co-treated with HG and PA with or without ALK7-shRNA were determined by western blot. *n* = 3 per group. *p < 0.05 vs. the control group, ^#^ *p* < 0.05 vs. the HG + PA + nc-shRNA group. **(E–F)**. The percentage of VSMC apoptosis treated by co-treatment of HG and PA with or without ALK7-shRNA was determined by TUNEL assay. *n* = 4 per group. Scale bar: 20 μm. The control group was added with 5.5 mM glucose, 19.5 mM mannose and 0.4 mM BSA. *p < 0.05 vs. the control group, ^#^ *p* < 0.05 vs. the HG + PA + nc-shRNA group. Data was analyzed with ANOVA.

In the late phase of atherosclerosis, increased apoptosis of VSMCs leads to thinner fibrillar cap, which is one major pathogenesis of plaque vulnerability. Therefore, we next determined to study the role of ALK7 on VSMC apoptosis. Results of *in vivo* experiments showed that ALK7 deficiency significantly increased the expression of Bcl-2 and decreased the expression of Bax in the diabetic group (Figures 4A,B). Besides, cleaved caspase 3 was reduced in the diabetic group with ALK7 knockdown (Figures 4A,B).

Then we added high glucose and palmitic acid to VSMCs to mimic diabetic atherosclerosis *in vivo* (Reddy et al., 2016) and found that co-treatment of high glucose and palmitic acid significantly upregulated the expression of ALK7, taking on time-dependent manner and being not influenced by the osmotic pressure (Supplementary Figure S2). Moreover, co-treatment of high glucose and palmitic acid induced obvious downregulation of Bcl-2 and upregulation of Bax and cleaved caspase 3 (Supplementary Figure S2).

In order to verify the role of ALK7 on high glucose and palmitic acid-induced VSMC apoptosis, we transfected VSMCs with ALK7 siRNA (Supplementary Figure S3) and found that ALK7 deficiency down-regulated the Bax and cleaved caspase-3 level and up-regulated the expression of Bcl-2 ((Figures 4C,D). Results of TUNEL staining showed similar results (Figures 4E,F). These findings suggest that ALK7-regulated VSMC apoptosis might be one key pathogenesis of plaque vulnerability in diabetes.

### Activin receptor-like kinase 7-Smad2/3 signaling pathway mediated high glucose and palmitic acid-induced vascular smooth muscle cells apoptosis

**FIGURE 5:**
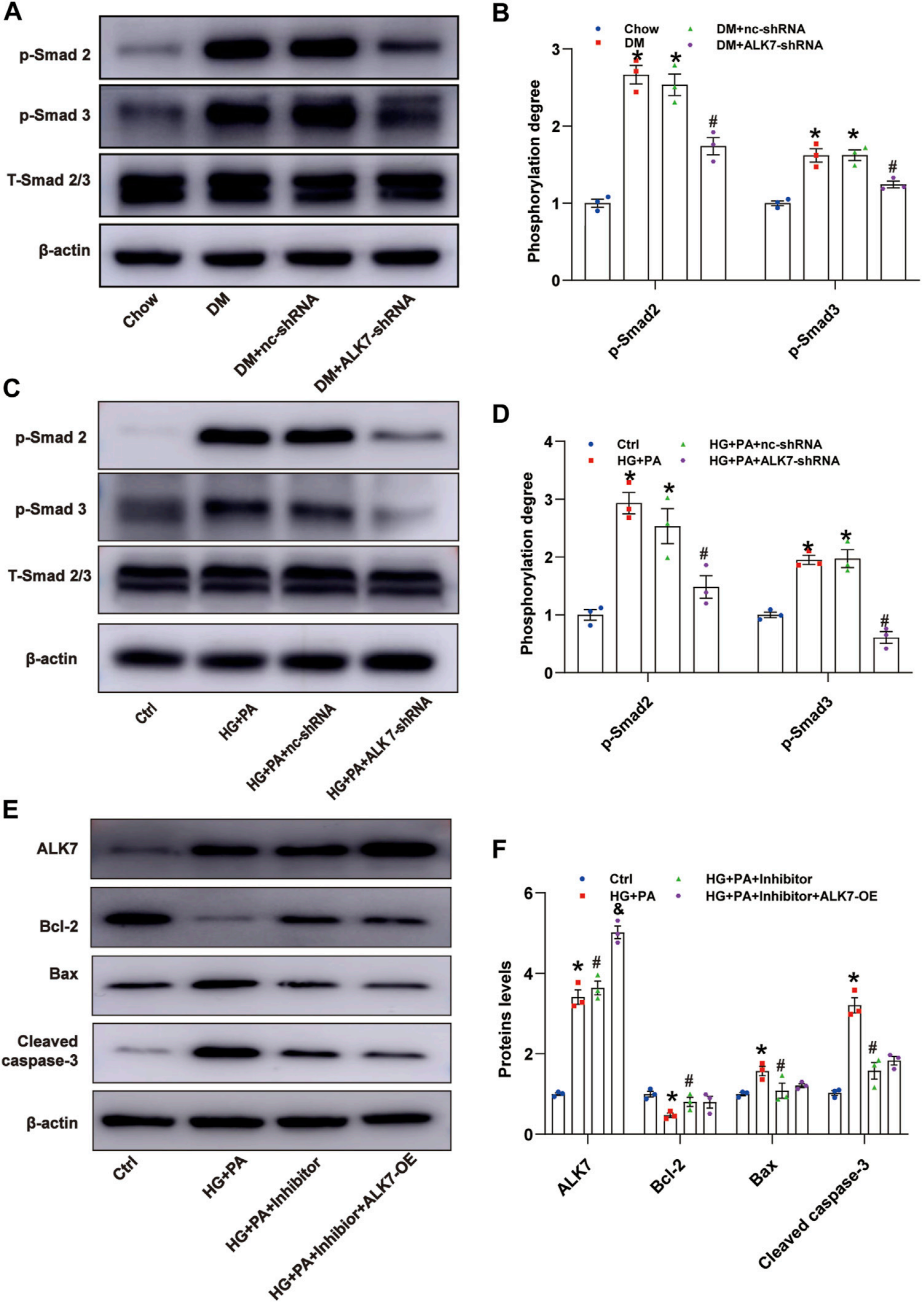
ALK7 promoted VSMC apoptosis *via* Smad2/3 signaling. **(A–B)**. The total and phosphorylation of Smad2 and Smad3 in the aortas with atherosclerotic plaques of diabetic mice were determined by western blot. *n* = 3 per group. *p < 0.05 vs. the Chow group, ^#^ *p* < 0.05 vs. the DM + nc-shRNA group. **(C–D)**. The total and phosphorylation of Smad2 and Smad3 in VSMCs co-treated with HG and PA were determined by western blot. *n* = 3 per group. *p < 0.05 vs. the control group, ^#^ *p* < 0.05 vs. the HG + PA + nc-shRNA group. **(E–F)**. The protein level of ALK7, Bcl-2, Bax, and cleaved caspase 3 in VSMCs co-treated with HG and PA and Smad2/3 inhibitor with or without ALK7-shRNA were determined by western blot. *n* = 3 per group. *p < 0.05 vs. the control group, ^#^ *p* < 0.05 vs. the HG + PA group, ^&^ *p* < 0.05 vs. the HG + PA + inhibitor. Data was analyzed with ANOVA.

Smad2 and Smad3 are known downstream molecules of ALK7 (Watanabe et al., 1999), and in this study, we determined to explore the role of Smad2/3 in ALK7-induced VSMC apoptosis. It was shown that phosphorylation of Smad2/3 was induced greatly in the diabetic group, which was reduced by the knockdown of ALK7 (Figures 5A,B). Similarly, high glucose and palmitic acid treatment obviously increased the phosphorylation of Smad2/3 in the VSMCs, which was efficiently reversed by ALK7 inhibition (Figures 5C,D). Moreover, application of Smad2/3 inhibitor significantly decreased VSMC apoptosis caused by high glucose and palmitic acid co-treatment (Figures 5E,F; Supplementary Figure S4). However, ALK7 overexpression exerted no significant impact on the inhibition of VSMC apoptosis by Smad2/3 inhibitor (Figures 5C,D).

## Discussion

In this study, we found that ALK7 was elevated in the atherosclerotic plaques of diabetic mice. Inhibiting ALK7 expression stabilized atherosclerotic plaques and reduced VSMC apoptosis. Furthermore, ALK7 exerted its pro-apoptotic role via Smad2/3 signaling pathway. Our findings indicated ALK7 might be a potential target for alleviating diabetic atherosclerosis.

The coexistence of diabetes aggravates the vulnerability of atherosclerotic plaques, resulting in the increased incidence and severity of clinical events, even death (Moreno and Fuster, 2004; Yuan et al., 2019; Xu et al., 2020). However, there are no effective therapies in this field. ALK7 was initially reported to play a crucial role in fat metabolism and other metabolic disturbance, such as diabetes (Ibáñez, 2021). The key role of ALK7 in diabetes and atherosclerosis respectively promoted us to further explore its role in diabetic atherosclerosis (Liu et al., 2013; Li et al., 2015; Ibáñez, 2021; Zhao et al., 2021). As expected, we found elevated ALK7 expression in the atherosclerotic plaques of diabetic mice and made clear that suppression of ALK7 could generate beneficial effects via inhibiting the progress of diabetic atherosclerosis, as indicated by the decreased plaque load and area along the aortic tree. Our findings fully testify ALK7 as an important modulator and potential therapeutic target in diabetic atherosclerosis.

There are different degrees of abnormal glucose and lipid metabolism in patients with type 2 diabetes mellitus (Gade et al., 2010). In the present study, we found that ALK7 exerted no significant effects on the level of serum lipids and glucose. Histological staining of aortic roots presented decreased index of vulnerable plaques in diabetic group with administration of ALK7-shRNA lentivirus. Therefore, we speculated ALK7 might exert its crucial impacts beyond metabolic regulation. Increased VSMC apoptosis brings about thinner fibrillar cap, destabilizes atherosclerotic plaques and promotes plaque rupture (Silva et al., 1995; Paneni et al., 2013). We innovatively demonstrated the promotion of ALK7 on VSMC apoptosis, suggesting ALK7 acted as a potential regulator of VSMCs. Recently, ALK7 was reported to promote VSMC phenotypic modulation induced by platelet-derived growth factor-BB via inhibiting PPARγ expression in the pathogenesis of intimal hyperplasia (Gong et al., 2020). Our findings about ALK7 in aggravating VSMC apoptosis of diabetic atherosclerosis expanded action of ALK7 on the biological characteristics of VSMCs.

The Smads family is a group of transcription factors that transmit TGF-beta-regulated signals directly from cell surface receptors to the nucleus, controlling a range of biological activities, such as proliferation, apoptosis, differentiation (Derynck et al., 1998; Zhou et al., 1999; Budi et al., 2017). Among them, Smad2/3 are receptor-activated Smad proteins that can be activated by the type I Activin receptors (ALK4/5/7) (Massagué, 1998) (Siegel and Massagué, 2003). In this study, the elevated phosphorylation of Smad2/3 in VSMCs co-cultured with high glucose and palmitic acid was significantly inhibited by ALK7 knockdown, indicating Smad2/3 as the downstream of ALK7. Moreover, ALK7 overexpression could not alleviate the anti-apoptotic effect of Smad2/3 inhibitors, further confirming that ALK7 regulated VSMCs apoptosis via Smad2/3 signaling. Our findings were consistent with other studies that ALK7 can regulate cell apoptosis through Smad2/3 (Xu et al., 2004; Zhao et al., 2012; Liu et al., 2013).

To sum up, our study demonstrated that interfering ALK7 expression significantly increased the stability of atherosclerotic plaques in ApoE^−/−^ mice with type 2 diabetes mellitus. Moreover, ALK7 was involved in the regulation of VSMC apoptosis via Smad2/3 signaling pathway, indicating ALK7 as a vital target in the treatment of diabetic atherosclerosis.

## Data Availability

The raw data supporting the conclusions of this article will be made available by the authors, without undue reservation.
