# Supplementary figures and images for: Activin receptor-like kinase 7 promotes apoptosis of vascular smooth muscle cells via activating Smad2/3 signaling in diabetic atherosclerosis
Source: Front Pharmacol. 2022 Aug 17;13:926433. doi: 10.3389/fphar.2022.926433 (PMC9428160; doi:10.3389/fphar.2022.926433)

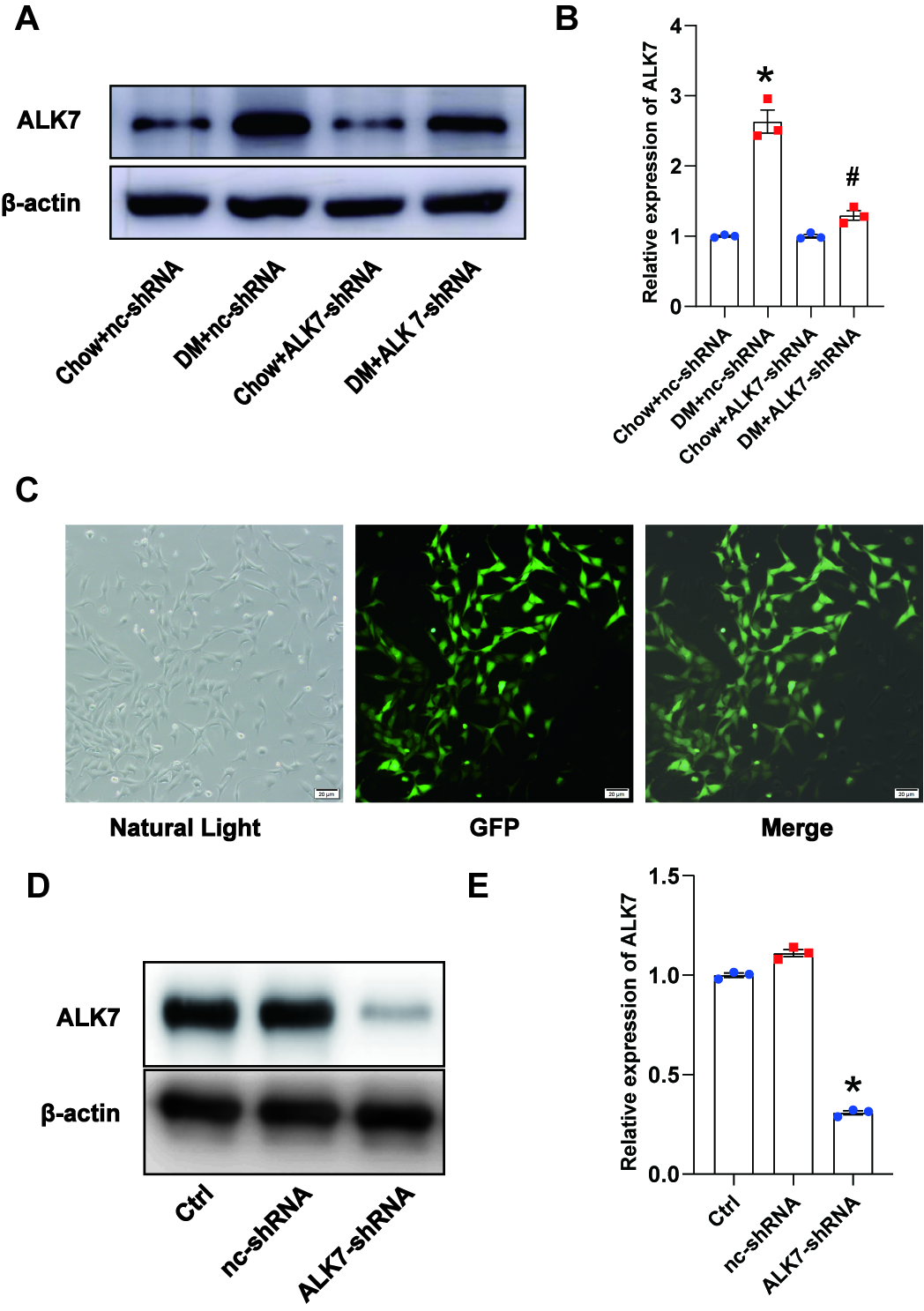

Supplement: Supplementary file 1 [file Image3.TIF]

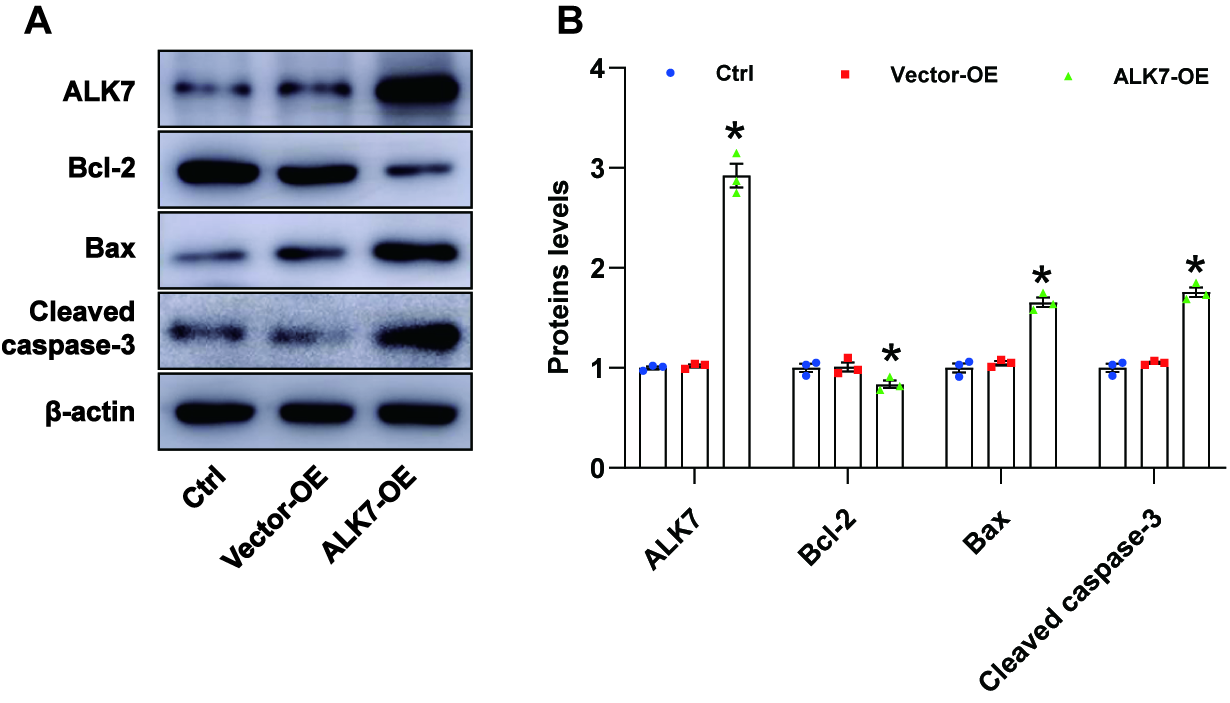

Supplement: Supplementary file 2 [file Image4.TIF]

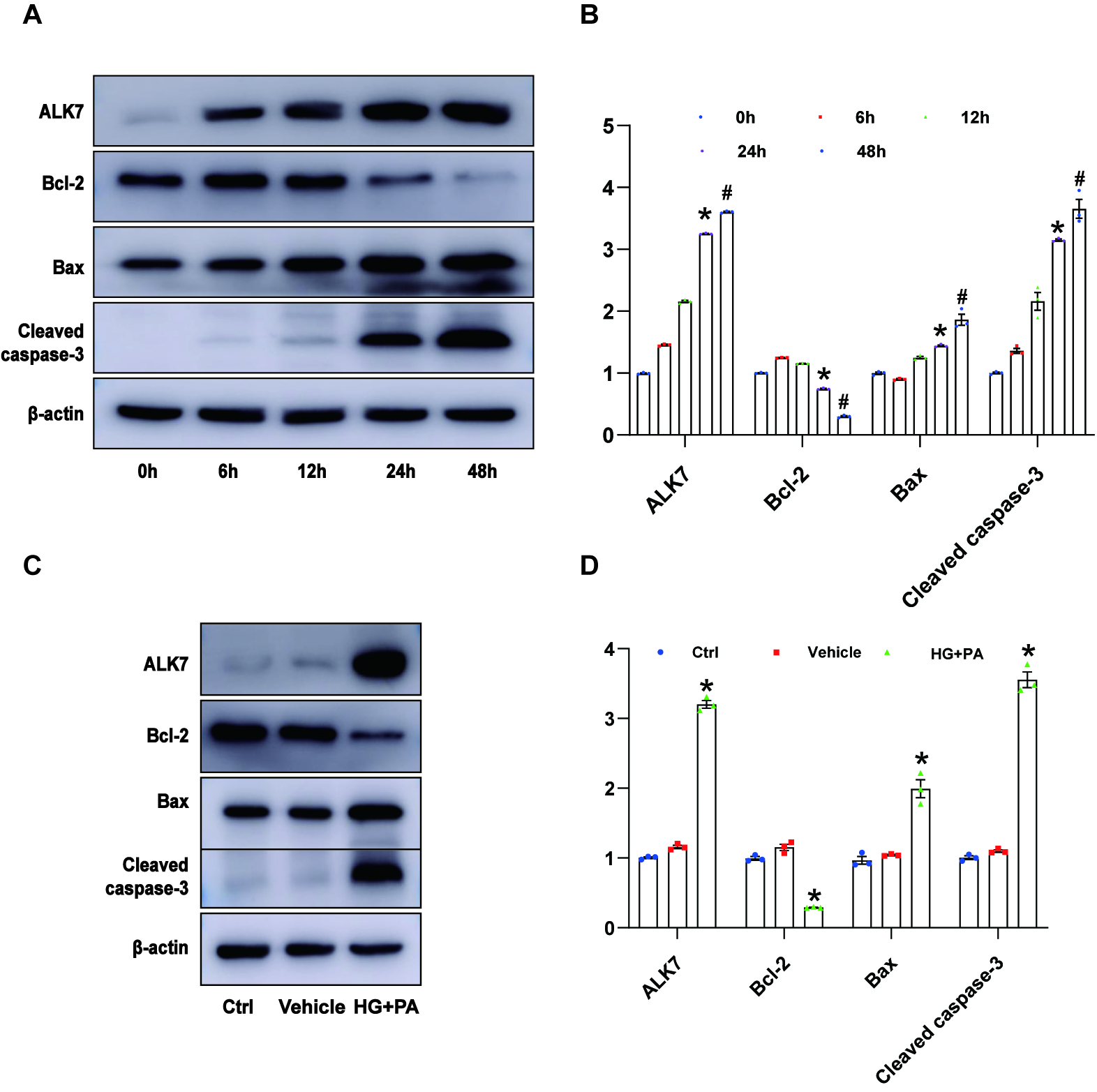

Supplement: Supplementary file 3 [file Image2.TIF]

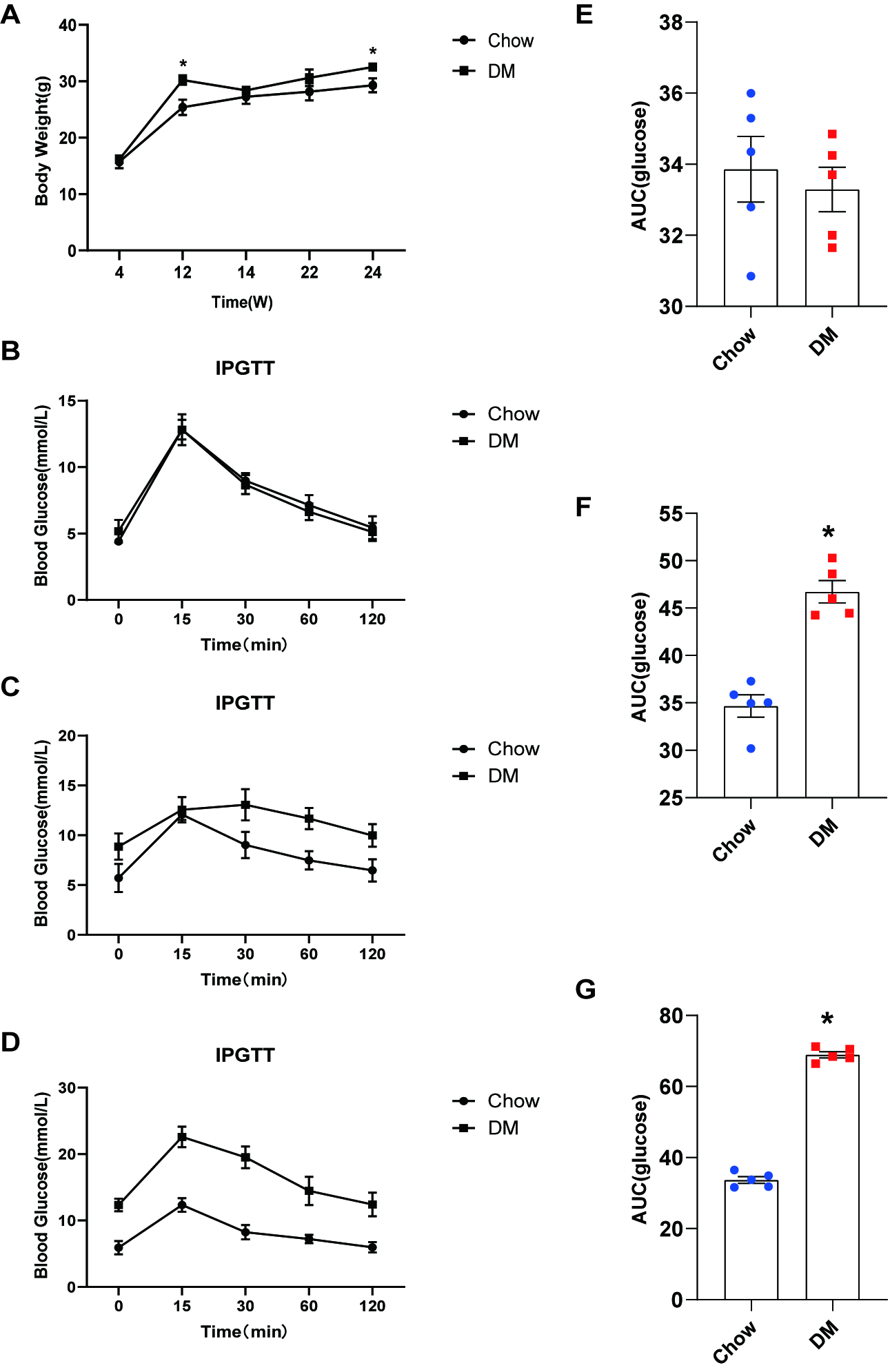

Supplement: Supplementary file 4 [file Image1.TIF]
